# Endoscopic surgery and temporalis muscle flap reconstruction for skull base osteoradionecrosis after radiotherapy

**DOI:** 10.3389/fonc.2026.1806033

**Published:** 2026-03-26

**Authors:** Feng Wang, Yanling Su, Liying Lin, Jianpeng Dai, Qilin Gong, Youyuan Shi, Lu Zhang

**Affiliations:** 1 Department of Head and Neck Surgery, Clinical Oncology School of Fujian Medical University, Fujian Cancer Hospital, Fuzhou, Fujian, China; 2 Department of Developmental and Behavioral Pediatrics, Fujian Children’s Hospital (Fujian Branch of Shanghai Children’s Medical Center), College of Clinical Medicine for Obstetrics & Gynecology and Pediatrics, Fujian Medical University, Fuzhou, Fujian, China; 3 Department of Operation, Clinical Oncology School of Fujian Medical University, Fujian Cancer Hospital, Fuzhou, Fujian, China

**Keywords:** endoscopic surgical procedure, nasopharyngeal carcinoma, quality of life, skull base osteoradionecrosis, temporal muscle flap

## Abstract

**Introduction:**

Skull base osteoradionecrosis (sbORN) is a serious late complication of radiotherapy for nasopharyngeal carcinoma (NPC), with limited effective treatment options. In this retrospective cohort study, we aimed to compare the outcomes and treatment efficacy between patients with sbORN who did and those who did not undergo endoscopic extended resection with temporalis muscle flap reconstruction.

**Methods:**

The study involved 57 patients (24 female and 33 male patients; age range: 41–78 years) diagnosed with sbORN following radiotherapy for NPC between September 2019 and September 2022 at our hospital, followed up until April 2025. The surgical group (n = 30) underwent extended endoscopic resection of necrotic skull base lesions with temporalis muscle flap reconstruction, whereas the non-surgical group (n = 27) received conservative management. Therapeutic interventions, demographic characteristics, survival, and symptomatic/quality-of-life profiles were evaluated.

**Results:**

In the surgical group, the mean pain and foul odor scores decreased significantly over 12 months, and the quality-of-life scores improved substantially over 12 months. The surgical group had a significantly longer median survival time and a higher 24-month overall survival rate than the non-surgical group did. Furthermore, multivariable analysis confirmed surgical intervention as an independent favorable prognostic factor for survival, which was associated with a reduced incidence of severe complications such as carotid blowout syndrome.

**Discussion:**

Endoscopic extended resection with temporalis muscle flap reconstruction appears to be a safe, effective, and feasible treatment for sbORN following radiotherapy for NPC.

## 1 Introduction

Nasopharyngeal carcinoma (NPC) is a malignant epithelial tumor that originates in the nasopharynx and is highly prevalent in Southeast Asia and South China (1, 2). The age-standardized incidence rate ranges from 3.0 per 100,000 in China to 0.4 per 100,000 in predominantly White populations (3). With the application of intensity-modulated radiotherapy, locoregional control has improved, increasing the clinical importance of late complications (4).

Skull base osteoradionecrosis (sbORN) is one of the most serious late complications of radiotherapy for NPC, and its main clinical symptoms include headache, foul odor, and epistaxis. This condition severely affects the quality of life (QoL) of patients and, in some cases, can lead to massive hemorrhage or death from intracranial infection (5). The incidence of nasopharyngeal and skull base necrosis after first-course radiotherapy for primary NPC is 2%–10% (6), and it may exceed 30% after re-irradiation for recurrent NPC (7, 8). The overall 2-year survival rate of patients with radiation-induced sbORN is less than 60%, with fatal massive hemorrhage due to internal carotid artery (ICA) rupture accounting for more than 90% of deaths (9). Primary treatment modalities, including hyperbaric oxygen therapy, administration of pentoxifylline with vitamin E, and local debridement, have demonstrated limited efficacy (10).

With advances in endoscopy and a better understanding of skull base anatomy, necrotic bone and soft tissue of the skull base can be completely removed. Repair and reconstruction using local, rotational, or free tissue flaps are critical for proper treatment. These techniques help protect important skull base structures, reduce the rate of nasopharyngeal hemorrhage and intracranial infection, and promote wound healing (11, 12).

Given the extensive nature of skull base defects, reconstruction requires flaps with substantial tissue volume and robust vascularity. Temporalis muscle flap reconstruction has been proven to be a reliable and effective method for reconstruction (13, 14).

Despite the severity of sbORN and the limited effectiveness of existing treatments, this condition has not received sufficient attention in clinical research. Since 2019, our center has performed endoscopic resection of necrotic skull base lesions combined with temporalis muscle flap reconstruction. Building on this experience, in the present study, we aimed to investigate the clinical manifestations and treatment outcomes of sbORN following radiotherapy for NPC with a focus on the effects of this surgical approach. Specifically, the objective of this study was to assess treatment efficacy by evaluating changes in the severity of pain and foul odor after surgery, and by comparing QoL complications and survival outcomes between patients who did and those who did not undergo endoscopic surgery combined with temporalis muscle flap reconstruction.

## 2 Materials and methods

### 2.1 Clinical data

This retrospective cohort study involved 57 patients with sbORN who were treated at Fujian Cancer Hospital between September 2019 and September 2022. All patients had previously received radiotherapy for NPC. To accurately characterize the baseline disease burden and extent of osteoradionecrosis, the severity of lesions for all patients was retrospectively evaluated and classified according to the grading system for post-radiation nasopharyngeal necrosis proposed by Zhang et al. (15). All surgical candidates underwent high-resolution computed tomography angiography and/or contrast-enhanced magnetic resonance angiography. For high-risk patients, multidisciplinary consultations, including those with the radiology and interventional departments, were also conducted, and balloon occlusion testing was performed when necessary. In this study, “invasion” of the ICA was defined as the presence of necrotic bone or inflammatory tissue involving more than 180° of the ICA circumference on imaging. Data on symptomatic profiles (pain/foul odor symptom severity in the surgical group), QoL, complications, and survival outcomes were collected. The protocol adhered to the principles of the Declaration of Helsinki and was approved by the Ethics Committee of Fujian Cancer Hospital (Approval No. K2025-082-01). Written informed consent was obtained from all patients for participation in the study and for the publication of this manuscript and any accompanying images.

### 2.2 Inclusion criteria

The general inclusion criteria for all patients were as follows: 1) a confirmed history of radiotherapy for NPC, with complete clinical data; 2) a diagnosis of cranial base radiation-induced bone necrosis confirmed based on symptoms, nasal endoscopic examination, imaging, and pathological biopsy results; and 3) willingness to comply with follow-up and absence of local recurrence or distant metastasis at the time of sbORN diagnosis (or at the time of surgery for the surgical group).

The additional criteria for the surgical subgroup were as follows: 1) the necrotic lesion being deemed resectable en bloc (completely removable) during the preoperative surgical planning, and 2) the surgical plan involving the use of a temporalis muscle flap and being successfully executed for reconstruction of the resultant defect.

Those who did not meet these criteria and those who declined surgery were assigned to the non-surgical group. For patients assigned to the non-surgical group, conservative management was tailored based on individual clinical presentations. The non-surgical treatment modalities primarily consisted of routine nasal endoscopic debridement and irrigation, systemic administration of broad-spectrum antibiotics for acute infections, hyperbaric oxygen therapy, and anti-fibrotic medications (e.g., pentoxifylline, vitamin E, and bisphosphonates).

### 2.3 Exclusion criteria

The exclusion criteria for all patients were as follows: 1) incomplete clinical data, 2) confirmation of tumor recurrence through preoperative biopsy or postoperative pathology, and 3) inability to complete the required follow-up for any reason.

The additional exclusion criteria for the surgical subgroup were as follows: incomplete removal of the necrotic lesion during surgery. These criteria were applied to evaluate the per-protocol efficacy of successfully executed reconstructions. However, to ensure transparency and address potential selection bias, the clinical outcomes of these initially excluded patients are reported separately in the results.

### 2.4 Surgical procedures

**Figure 1:**
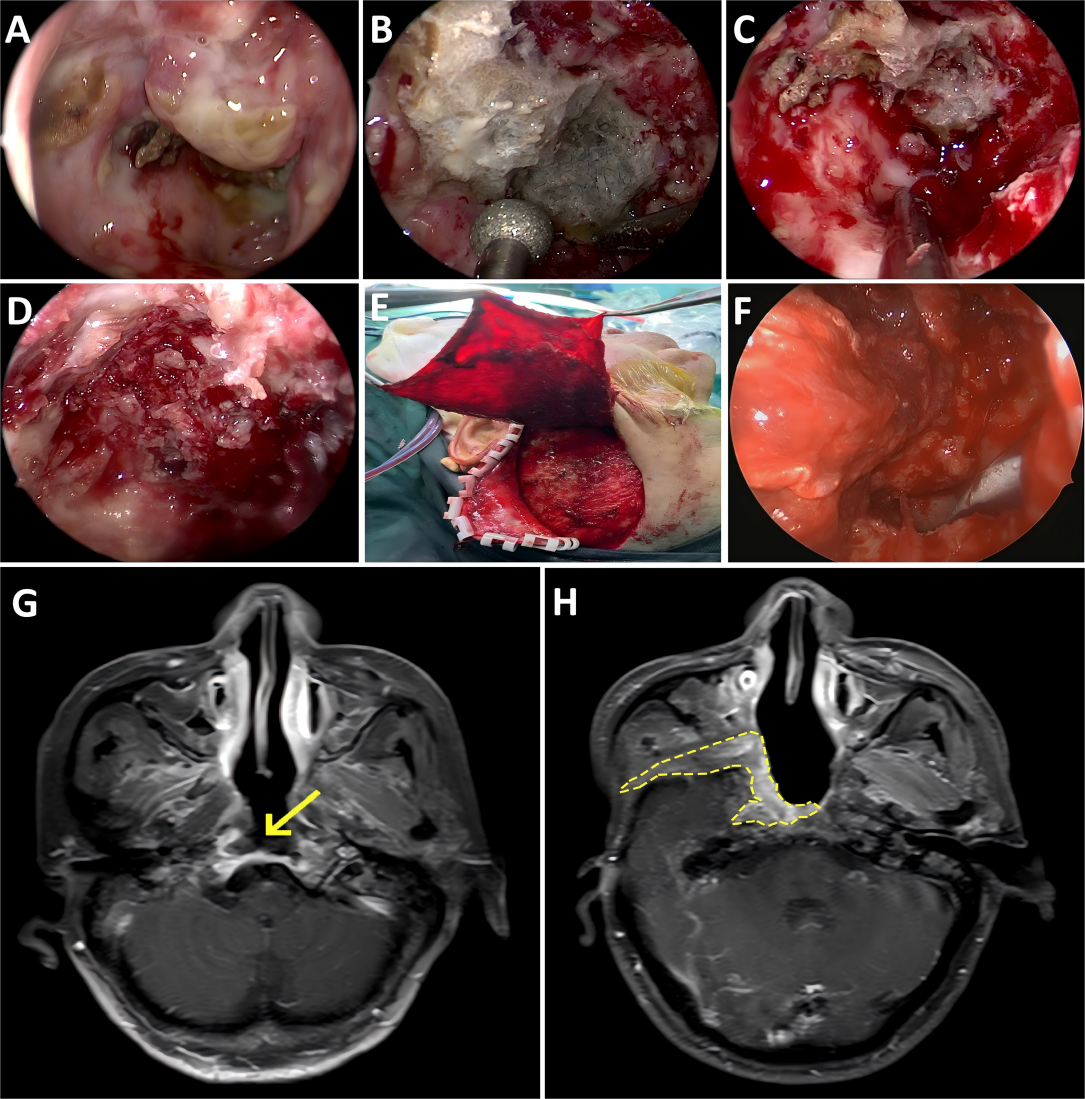
Surgical procedures and magnetic resonance images. **(A)** Necrotic ulcers in the nasopharynx. **(B, C)** Removal of bone necrosis at the base of the skull using a drill. **(D)** Fresh bone at the base of the skull. **(E)** Temporal muscle flap. **(F)** Repair of the skull base wound using a temporal muscle flap. **(G)** Preoperative image: The arrow points to the nasopharyngeal ulcer accompanied by skull base necrosis. **(H)** Postoperative image: The yellow dotted line indicates that the temporal muscle flap had survived.

Typically, an extended lateral nasal wall approach was employed, which involves removal of the uncinate process and opening of the maxillary sinus, followed by superior reflection of the inferior turbinate along the lateral nasal wall. The pterygopalatine fossa and pterygoid structures were laterally displaced or resected to expose the lesion and its lateral boundaries. The posterior segment of the nasal septum was removed, and the floor and anterior wall of the sphenoid sinus were resected to reveal the contralateral boundary and clival lesions. The lesion was excised with adequate margins demarcated by viable skull base bone or freshly vascularized muscle tissue. Exceptional care was taken during debridement adjacent to the ICA. Macroscopic necrotic bone and inflammatory granulation tissue were radically resected; however, if necrotic tissue densely adhered to the ICA adventitia, a microscopic thin layer was deliberately preserved to avoid iatrogenic vascular rupture. Subsequently, an incision was made extending from 2 cm superior to the lateral supraorbital margin along the superior temporal line to the temporo-occipital junction, turning toward the superior auricular border and anteriorly along the zygomatic arch to the pretragal area. The flap was elevated deep into the superficial temporal fascia in an anteroinferior direction until it reached the superior border of the superficial temporal fat pad, where the deep temporal fascia was incised to expose the temporalis muscle fibers. The superficial temporal fat pad, along with the superficial temporal fascia, subcutaneous tissue, and skin, was reflected superiorly along a plane deep within the deep temporal fascia, separating the zygomatic arch from the inferior portion of the temporalis muscle. The temporalis muscle attachments were divided along the skin incision and lateral orbital rim. The periosteum, temporalis muscle, and deep temporal fascia were subperiosteally elevated from the temporal bone surface to the infratemporal fossa to form a pedicled temporalis muscle flap, based on the deep temporal vascular pedicle with a pivot at the mandibular coronoid process. The distal end of the muscle flap was transposed into the surgical field through the infratemporal and pterygopalatine fossa. The flap was positioned to cover the defect, with its deep temporal fascial surface facing the nasopharynx. A gelatin sponge and iodoform gauze packing were applied for compression and secured using a Foley catheter balloon. A drain was placed at the temporal donor site, followed by layered wound closure and the application of a pressure dressing (**Figures 1A–F**).

### 2.5 Follow-up protocol

For patients in the surgical group, postoperative assessments included nasoendoscopy and magnetic resonance imaging (including vascular sequences when necessary, to monitor the integrity of the ICA and flap viability) performed every 3 months (**Figures 1G, H**). Additionally, the surgical group was assessed for pain and foul odor symptom severity (numeric rating scale [NRS] scores), along with the QoL (European Organization for Research and Treatment of Cancer Quality of Life Questionnaire, version 3.0) at 1, 3, 6, and 12 months via telephone interview or outpatient follow-up. These specific assessments (nasoendoscopy, magnetic resonance imaging, and pain and foul odor severity assessments) were not performed for the non-surgical group owing to a different clinical follow-up protocol. For the non-surgical group, the follow-up primarily focused on survival outcomes and QoL, with the QoL data systematically collected at 1, 3, 6, and 12 months after the diagnosis of sbORN. Patient survival status was documented monthly for all patients from the date of sbORN diagnosis.

### 2.6 Statistical analysis

Following data collection and collation, analyses were performed using IBM SPSS Statistics for Windows, version 22.0 (IBM Corp., Armonk, NY, USA), and GraphPad Prism (version 9.5.1; GraphPad Software, San Diego, CA, USA). Categorical variables were presented as frequency and percentage. Continuous variables were expressed as the mean ± standard deviation or median (interquartile range) depending on their distribution. Continuous variables were analyzed using independent-samples *t*-tests after assessing normality, whereas non-parametric tests were used when distributional assumptions were not met. Repeated-measures data were evaluated using a mixed linear model. Survival analysis was performed using Kaplan–Meier curves. To adjust for potential confounders arising from non-randomized treatment allocation, a multivariable Cox proportional hazards regression model was additionally performed to calculate hazard ratios (HRs) and 95% confidence intervals (CIs) for key prognostic covariates. Statistical significance was set at *p* < 0.05.

## 3 Results

### 3.1 Clinical characteristics

We retrospectively analyzed the data of 57 patients who developed sbORN following radiotherapy for NPC at our institution from September 2019 to September 2022. The surgical (n = 30) and non-surgical (n = 27) groups were well-matched, with no significant differences in sex, age, number of previous radiotherapy sessions, ICA invasion status, baseline symptom burden (pain and foul odor NRS scores), or baseline disease severity (post-radiation nasopharyngeal necrosis grade; all *p* > 0.05). The detailed demographic and clinical profiles are presented in **Table 1**. During the study period, one additional patient initially underwent the surgical procedure but was excluded from the primary 30-patient surgical cohort. In this patient, complete resection could not be achieved because of a high intraoperative risk of bleeding; the patient subsequently developed a severe local infection and died within 6 months.

**Table 1:** Patient characteristics.

Characteristic	Surgical group (n = 30)	Non-surgical group (n = 27)	*p* value
Sex			0.38
Male	19 (63.3%)	14 (51.9%)	
Female	11 (36.7%)	13 (48.1%)	
Age (y)			0.30
≥ 55	21 (70.0%)	23 (85.2%)	
< 55	9 (30.0%)	4 (14.8%)	
Number of radiotherapy sessions			0.66
1	23 (76.7%)	22 (81.5%)	
2	7 (23.3%)	5 (18.5%)	
Invasion of the ICA^†^			0.73
Yes	18 (60.0%)	15 (55.6%)	
No	12 (40.0%)	12 (44.4%)	
Pain NRS score			0.24
≥ 7	25 (83.3%)	19 (70.4%)	
< 7	5 (16.7%)	8 (29.6%)	
Foul odor NRS score			0.57
≥ 5	21 (70.0%)	17 (63.0%)	
< 5	9 (30.0%)	10 (37.0%)	
Time from radiotherapy to sbORN (mo)	25.5 (17.0–46.0)	28.0 (19.0–61.0)	0.26
Baseline PRNN grade			0.92
I	0 (0%)	0 (0%)	
II	2 (6.7%)	3 (11.1%)	
III	10 (33.3%)	9 (33.3%)	
IV	18(60.0%)	15 (55.6%)	
Non-surgical treatments received^*^			N/A
Nasal endoscopic debridement/irrigation	N/A	27 (100%)	
Antibiotic therapy	N/A	27 (100%)	
Hyperbaric oxygen therapy	N/A	8 (30%)	
Pentoxifylline	N/A	4 (15%)	
Vitamin E	N/A	4 (15%)	
Bisphosphonates	N/A	0 (0%)	

ICA, internal carotid artery; NRS, Numeric Rating Scale; PRNN, post-radiation nasopharyngeal necrosis; N/A, not available.

^*^Patients in the non-surgical group may have received more than one type of conservative treatment.

^†^Invasion of the ICA was defined as the presence of necrotic bone or inflammatory tissue involving more than 180° of the ICA circumference on imaging.

### 3.2 Pain and odor severity

Patients who underwent surgery experienced a significant reduction in pain and foul odor symptom severity based on the preoperative versus postoperative NRS assessments at 1, 3, 6, and 12 months. The pain NRS scores decreased from 7.80 ± 1.32 preoperatively to 1.08 ± 0.81 at 12 months postoperatively (*p* < 0.001), and the foul odor NRS scores declined from 5.23 ± 1.36 preoperatively to 1.20 ± 1.56 at 12 months postoperatively (*p* < 0.001). These results confirmed significant symptomatic relief following surgery (**Table 2**).

**Table 2:** Preoperative and postoperative numeric rating scale scores (NRS) for pain and foul odor.

	Preoperative	1 month	3 months	6 months	12 months	*p* value
No. of patients	30	30	28	27	25	
Pain NRS score	7.80 ± 1.32	3.93 ± 1.72	2.75 ± 1.11	1.74 ± 1.06	1.08 ± 0.81	< 0.001
Foul odor NRS score	5.23 ± 1.36	3.03 ± 2.14	1.46 ± 1.48	1.11 ± 1.50	1.20 ± 1.56	< 0.001

### 3.3 QoL

**Figure 2:**
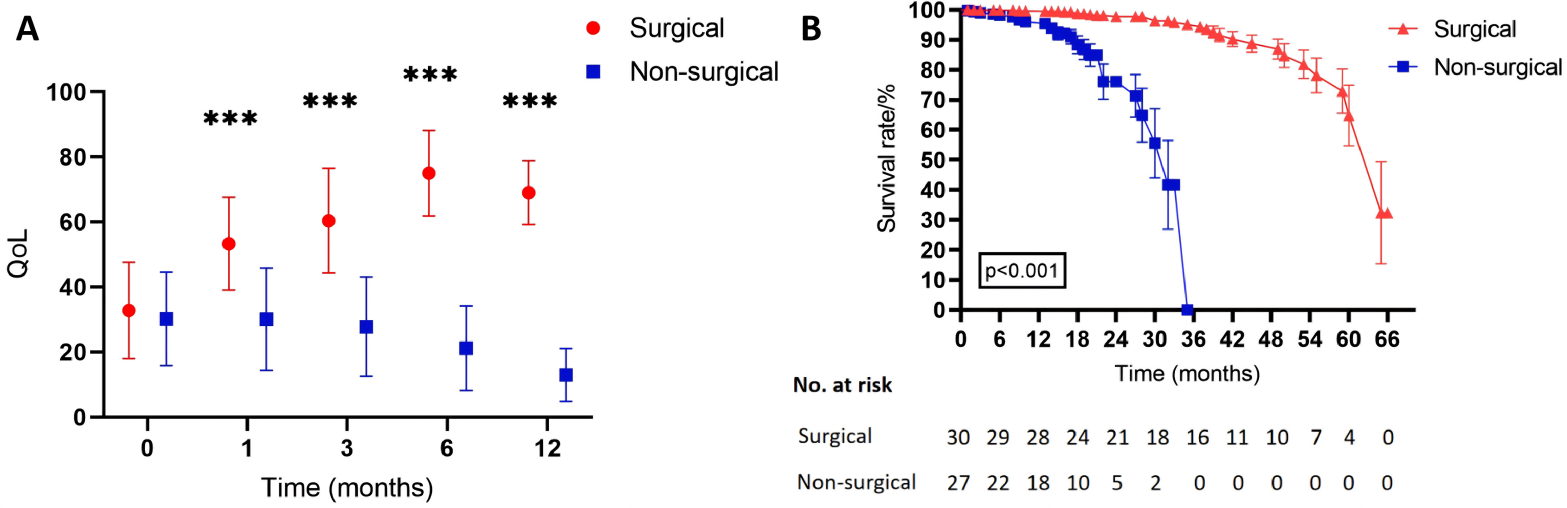
Comparison of QoL and survival between the surgical and the non-surgical groups. **(A)** Comparison of QoL scores over 12 months. The QoL score of the surgical group is significantly higher than that of the non-surgical group. **(B)** Kaplan–Meier curve comparing long-term survival. The surgical group demonstrates a significantly higher survival rate than the non-surgical group does, throughout the follow-up period. QoL: quality of life; ***p < 0.001.

The global health status/QoL domain of the European Organization for Research and Treatment of Cancer Quality of Life Questionnaire was used to evaluate QoL. Patients in the non-surgical group exhibited progressive deterioration (prediagnosis: 30.2 ± 14.3; 12 months postdiagnosis: 12.96 ± 8.2). Conversely, patients in the surgical group demonstrated a significant improvement (preoperative: 32.8 ± 14.8; 12 months postoperative: 69.0 ± 9.8). Between-group comparisons reached statistical significance from the first postoperative month (all *p* < 0.001), despite comparable baseline scores (*p* = 0.60). The therapeutic benefit increased over time, peaking at 6 months (surgical group: 75.0 ± 13.1 vs. non-surgical group: 21.2 ± 13.0; Δ = 53.8 points; **Table 3** and **Figure 2A**).

**Table 3:** Comparison of the quality of life scores between the surgical and non-surgical groups.

Group		Preoperative or prediagnosis	1 month	3 months	6 months	12 months
Non-surgical	Mean ± SD	30.2 ± 14.3	30.1 ± 15.7	27.8 ± 15.3	21.2 ± 13.0	12.96 ± 8.2
	N	27	26	24	22	18
Surgical	Mean ± SD	32.8 ± 14.8	53.3 ± 14.3	60.4 ± 16.1	75.0 ± 13.1	69.0 ± 9.8
	N	30	30	28	27	25
*p* value		0.60	< 0.001	< 0.001	< 0.001	< 0.001

SD, standard deviation; N, number of patients.

### 3.4 Survival

The survival analysis showed a significantly longer median survival time in the surgical group than in the non-surgical group (50 months, 95% confidence interval: 32.2–67.7 vs. 15 months, 95% confidence interval: 9.9–20.1; log-rank *p* < 0.001). Additionally, the 24-month overall survival (OS) rate was markedly higher in the surgical group than in the non-surgical group (63.3% vs. 18.5%) (**Figure 2B**). This survival benefit was closely related to the incidence of major life-threatening complications. During follow-up, carotid blowout events occurred in 2 patients (6.7%) in the surgical group, compared with 5 patients (18.5%) in the non-surgical group. Similarly, severe intracranial infections developed in 1 patient (3.3%) in the surgical group versus 6 patients (22.2%) in the non-surgical group.

Given the non-randomized nature of treatment allocation, a multivariable Cox regression analysis was performed to adjust for potential confounders (**Table 4**). After adjusting for age, sex, number of radiotherapy sessions, ICA invasion, and time from radiotherapy to sbORN, surgical intervention remained an independent favorable prognostic factor for OS (HR = 0.14, 95% CI: 0.06–0.35, *p* < 0.01). Furthermore, a longer time interval from radiotherapy to sbORN diagnosis was also identified as a significant protective factor (HR = 0.96, 95% CI: 0.93–0.99, *p* = 0.01).

**Table 4:** Multivariable Cox proportional hazards regression analysis for overall survival.

Variables	HR	95% CI	*p* value
Treatment modality			
Non-surgical management	Reference		
Surgical intervention	0.14	0.06–0.35	< 0.01
Age (y)	1.01	0.97–1.04	0.77
Sex			
Male	Reference		
Female	0.54	0.26–1.13	0.10
Number of radiotherapy sessions			
1	Reference		
2	1.56	0.70–3.47	0.28
Invasion of the ICA^†^			
Yes	Reference		
No	0.62	0.24–1.56	0.31
Time from radiotherapy to sbORN (mo)	0.96	0.93–0.99	0.01

ICA, internal carotid artery; sbORN, skull base osteoradionecrosis; HR, hazard ratio; CI, confidence interval.

^†^Invasion of the ICA was defined as the presence of necrotic bone or inflammatory tissue involving more than 180° of the ICA circumference on imaging.

### 3.5 Complications and adverse events

A structured comparison of adverse events is presented in **Table 4**. For early postoperative safety in the surgical group, complications were predominantly Clavien–Dindo grade I/II (27 out of 30 patients). Three patients experienced partial flap necrosis requiring surgical intervention (grade III), and no life-threatening events (grade IV/V) occurred.

Long-term outcomes demonstrated that, compared with non-surgical management, surgical intervention significantly reduced the incidence of fatal or severe follow-up events, specifically carotid blowout syndrome (2 vs. 9, *p* = 0.03), cerebrospinal fluid leakage (1 vs. 7, *p* = 0.04), and recurrent epistaxis (6 vs. 14, *p* = 0.01). The incidence of systemic complications, such as pulmonary infection, did not differ between the cohorts (all *p* > 0.05; **Table 5**).

**Table 5:** Structured reporting of adverse events and complications.

Complications/adverse events	Surgical group (n = 30)	Non-surgical group (n = 27)	*p* value
Intraoperative complications			
Major bleeding	0	N/A	–
Cranial nerve deficits	3	N/A	–
Postoperative/follow-up events			
Carotid blowout syndrome	2	9	0.03
Severe intracranial infection	1	6	0.08
Cerebrospinal fluid leak	1	7	0.04
Recurrent epistaxis	6	14	0.01
Pulmonary infection	4	2	0.77
Deep vein thrombosis/pulmonary embolism	1	0	1.00
Partial flap necrosis/dehiscence	3	N/A	–
Highest Clavien–Dindo grade^*^ (surgical group)			
I/II (minor, medical therapy)	27	N/A	–
III (requiring surgical intervention)	3	N/A	–
IV/V (Life-threatening/death)	0	N/A	–

N/A, not available.

^*^The Clavien–Dindo classification was applied to evaluate early postoperative complications occurring within 30 days after surgery.

## 4 Discussion

sbORN is one of the most severe late complications of radiotherapy for NPC. It is characterized by marked tissue destruction, challenging clinical management, and high disability rates. The primary symptoms include intractable pain, foul nasal odor, and recurrent hemorrhage; in advanced cases, life-threatening complications, such as ICA rupture or intracranial infection, may occur (16). Critically, studies have confirmed that nasopharyngeal necrosis as an independent prognostic factor for OS in patients without metastasis, particularly those with ICA exposure who forgo surgical intervention and exhibit significantly poorer outcomes (17, 18). To address this refractory condition, we retrospectively analyzed the data of 57 patients with sbORN, of whom 30 underwent endoscopic extended resection with temporalis muscle flap reconstruction and 27 received non-surgical management. The results demonstrated that, compared with non-surgical management, temporalis muscle flap reconstruction not only achieved rapid and significant alleviation of pain and foul odor alongside substantial QoL improvement but also markedly prolonged the median survival time and enhanced the OS rate. Collectively, these findings provide robust evidence supporting surgical intervention as an effective therapeutic strategy for sbORN. Notably, our multivariable analysis confirmed surgical intervention as a strong independent protective factor for survival, even after adjusting for key baseline confounders. Furthermore, a longer latency from radiotherapy to sbORN was a favorable prognostic factor, likely reflecting a more gradual and less aggressive radionecrotic process.

Conventional treatment modalities for osteoradionecrosis include hyperbaric oxygen therapy and pharmacological interventions, such as antibiotics and anti-fibrotic medications (e.g., pentoxifylline, vitamin E, and bisphosphonates) (10, 19). However, their efficacy remains limited for deep-seated skull base lesions, as they often fail to adequately control infection, debride necrotic tissue, and promote healing, leading to an exceedingly poor prognosis. In the present study, the non-surgical group exhibited a median survival period of only 15 months, which aligns with the findings of previous studies and underscores the severity of sbORN and the urgent need for more effective therapeutic strategies (14, 20). Although open surgery via craniofacial approaches allows for radical debridement, it is associated with significant trauma, carries high risks in previously irradiated tissues, and is often compromised by poor healing potential, possibly resulting in new functional impairments and facial deformities. Free flap transplantation can ensure robust vascularity; however, it is technically demanding, requires prolonged operative time, causes donor-site morbidity, and carries a high risk of failure, particularly in the context of compromised recipient vessels in irradiated fields (21–23).

Our results suggest that surgical intervention may substantially improve symptoms and survival compared with non-surgical management. The key to its success lies in two core links: thorough debridement and vascularized tissue reconstruction. With advancements in endoscopic technology and an improved understanding of skull base anatomy, our center can completely resect necrotic bone and soft tissue of the skull base via surgery and innovatively utilize temporalis muscle flap repair. This approach effectively protects critical skull base structures, reduces the risk of massive epistaxis and intracranial infection, and accelerates the healing of skull base wounds (11, 12). Endoscopic surgery provides a direct, high-resolution visualization pathway to access the skull base region (24). This avoids the extensive soft tissue dissection and bone osteotomy that are required in traditional open surgery, significantly reducing surgical trauma. High-definition endoscopy magnifies the surgical field, enabling surgeons to precisely identify the boundary between necrotic and healthy tissues, maximize thorough removal of inflammatory granulation tissue and sequestrum, and simultaneously markedly reduce the risk of iatrogenic injury to the surrounding important nerves and blood vessels (25, 26).

The fundamental reason for the poor healing of sbORN is local tissue ischemia and hypoxia, whereas the temporalis muscle flap, with its constant and reliable axial vascular pedicle, features a rich blood supply and strong anti-infective capacity. Transferring the temporalis muscle flap to the debrided skull base space not only fills dead spaces and separates intracranial and extracranial communication, but also significantly improves local microcirculation with its robust pro-angiogenic capacity, directly promoting granulation tissue growth and bone healing. Compared with free flap transfer, pedicled temporalis muscle flap transfer is relatively simple, requiring no complex microvascular anastomosis and avoiding the risk of anastomotic failure in free flaps when recipient-site vascular conditions are poor. These factors may help reduce the incidence of severe complications, such as cerebrospinal fluid leakage, intracranial infection, and ICA rupture (27, 28). Indeed, our comparative analysis of major complications provided critical clinical validation for this safety profile. Early postoperative complications in the surgical group were systematically evaluated using the Clavien–Dindo classification. Although three patients experienced partial flap necrosis requiring secondary intervention (grade III), the procedure was generally well-tolerated. More importantly, the surgical group demonstrated a notably lower incidence of lethal or severe follow-up events—specifically, carotid blowout syndrome, cerebrospinal fluid leakage, recurrent epistaxis, and severe intracranial infections—compared with the non-surgical group. By providing a robust, well-vascularized physical barrier over the exposed ICA and sealing skull base defects, this surgical strategy effectively mitigates the risk of catastrophic vascular and infectious events, which are the primary drivers of the high mortality and poor survival observed in conservative management.

Notably, no internationally standardized treatment consensus or guidelines for sbORN have been established. Our study results demonstrate that patients in the surgical group achieved rapid and sustained relief from subjective symptoms, such as pain and foul odor, with their QoL and survival period being superior to those in the non-surgical group, underscoring the comprehensive value of endoscopic surgery combined with temporalis muscle flap reconstruction in the management of sbORN. Therefore, we believe that this surgical approach can serve as a critical treatment option for such patients by integrating minimal invasiveness, safety, and efficacy.

Owing to its retrospective design and single-center nature, this study has some inherent limitations, which may limit the generalizability of our findings. First, our primary survival and QoL analyses utilized a per-protocol approach by excluding one patient with incomplete resection. Although this demonstrates the optimal therapeutic benefit of a successfully completed procedure, it introduces a post-treatment selection bias that may overestimate the overall safety and efficacy, compared with an intention-to-treat analysis. To mitigate this, we have reported the specific outcomes in the patient in whom the resection was incomplete. Limitations also include a small cohort size and the lack of comparative data on other treatment modalities, such as open surgeries, alternative reconstruction methods, and systemic medical therapies. Consequently, our findings require further validation through prospective, large-scale, multicenter studies with long-term follow-up. Additionally, this surgical approach requires considerable technical expertise, has a substantial learning curve, and presents challenges for widespread implementation.

Based on our clinical experience and data obtained from this retrospective study, endoscopic endonasal radical resection of necrotic skull base lesions combined with temporalis muscle flap reconstruction appears to be a safe and feasible treatment approach for sbORN, as it was associated with improved symptoms and survival rates in this cohort of patients with radiation-induced sbORN following NPC radiotherapy. This technique leverages the minimally invasive advantages of endoscopic surgery to access deep skull base lesions while also using the temporalis muscle flap as a pedicled vascularized tissue flap, providing reliable soft tissue coverage and a physical barrier. In our cohort, this technique enabled thorough lesion clearance, enhanced control of infection and skull base-related complications, improved QoL, and prolonged survival. This surgical technique is a promising treatment option for refractory diseases. However, the definitive long-term efficacy and broader applicability potential of the treatment approach require further validation and optimization through future prospective large cohort studies and long-term follow-up.

## Data Availability

The raw data supporting the conclusions of this article will be made available by the authors, without undue reservation.
